# Acquired reactive perforating collagenosis: A case report and review of the literature

**DOI:** 10.1097/MD.0000000000020391

**Published:** 2020-05-29

**Authors:** Xinyue Zhang, Yan Yang, Shiying Shao

**Affiliations:** a Division of Endocrinology, Tongji Hospital, Huazhong University of Science and Technology; b Taikang Tongji (Wuhan) Hospital, Wuhan, P.R. China.

**Keywords:** diabetes mellitus, perforating dermatoses, reactive perforating collagenosis

## Abstract

**Introduction:**

Acquired reactive perforating collagenosis (ARPC) is a rare skin disorder, which is associated with various internal diseases and even malignant neoplasms. A comprehensive knowledge of the concomitant diseases in ARPC patients is helpful to decrease the misdiagnosis. Although the treatment of ARPC is challenging, systemic assessment of existing regimens is not available.

**Patient concerns:**

A 50-year-old woman was admitted to the hospital due to cutaneous pruritus and papules all over the body.

**Diagnosis:**

Physical examination showed various sized papules on the lower limbs, buttocks, back, chest, and upper arms with keratotic plugs in the center. Histopathology showed typical collagenous fiber perforation. The diagnosis of ARPC was made according to histopathology, onset age and typical skin lesions. Type 2 diabetes mellitus (T2DM), chronic renal failure (CRF), and hypothyroidism simultaneously presented in this patient.

**Interventions:**

This patient was initially treated with topical corticosteroids and oral antihistamines for the skin lesion and pruritus. Medications for glucose control and recovery of renal and thyroid functions were also applied. On the second admission, the combined therapy of topical retinoic acid, Chinese medicinal herb-Qingpeng ointment, and Zinc oxide ointment was added.

**Outcomes:**

Papules and pruritus were improved significantly after the second hospitalization.

**Conclusion:**

We present a case of ARPC associated with T2DM, CRF, and hypothyroidism, which has rarely been described. There is no standardized treatment for ARPC. Co-administration of two or more agents for dermatologic interventions and treatment for associated diseases may help to improve skin symptoms.

## 1 Introduction

Reactive perforating collagenosis (RPC), a subtype of perforating dermatoses (PD), is a rare skin disease, characterized by transepidermal elimination of altered dermal collagen.^[1]^ There are two types of RPC, acquired RPC (ARPC) and inherited RPC (IRPC).^[2]^ IRPC is more common in infants and children; while ARPC usually develops in adulthood in association with a variety of systemic disorders, such as type 2 diabetes mellitus (T2DM), chronic renal failure (CRF), hypertension, and even malignant neoplasms.^[2]^

Here, we reported for the first time to the best of our knowledge, a rare case of a 50-year-old woman who presented ARPC associated with three different systemic diseases including T2DM, CRF, and hypothyroidism. Thorough understanding of the associated systemic diseases and the concomitant incidence in ARPC patients is of significance to make a proper screening and decrease the misdiagnosis. In addition, the treatment of ARPC can be challenging. However, there is no standardized therapeutic strategy by now. Summarization and evaluation of existing regimens are necessitated.

## 2 Consent

This study was approved by the ethics committee of Tongji Hospital, Huazhong University of Science and Technology. Patient has provided informed consent for publication of the case.

## 3 Case report

A 50-year-old Chinese woman was admitted to our hospital with a chief complaint of cutaneous pruritus and poor blood glucose control. Seven years ago, she had pruritus on her lateral and anterior thighs without any skin lesions. Thereafter, the pruritus and lesions exacerbated, spreading all over the body. Two years ago, the patient was diagnosed as T2DM and received insulin therapy for glucose control.

Laboratory examinations showed elevated HbA1c level (6.3%), potassium (5.72 mmol/L), creatinine (150 μmol/L); and decreased eGFR (34.7 mL/min/1.73 m^2^) (Table 1). Urinary microalbumin reached 2891.5 mg/L. Arterial blood gas analysis revealed metabolic acidosis with PH value 7.263. Hypothyroidism was also observed in this patient (Table 1). The titers of anti-dsDNA antibodies and antinuclear antibodies were within normal range. Fundoscopy indicated obvious bleeding and exudation. Chest CT showed pulmonary infections in middle lobe of right lung, upper lobe of left lung and bilateral lower lobe. There were no clinical or biological clues of malignant diseases.

**Table 1:**
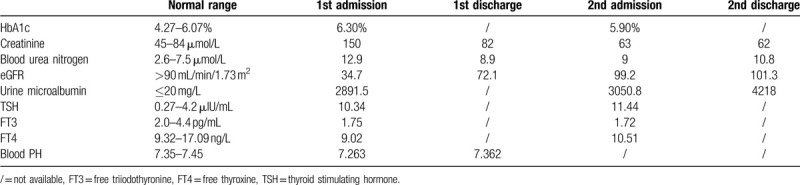
Laboratory examinations.

**Figure 1:**
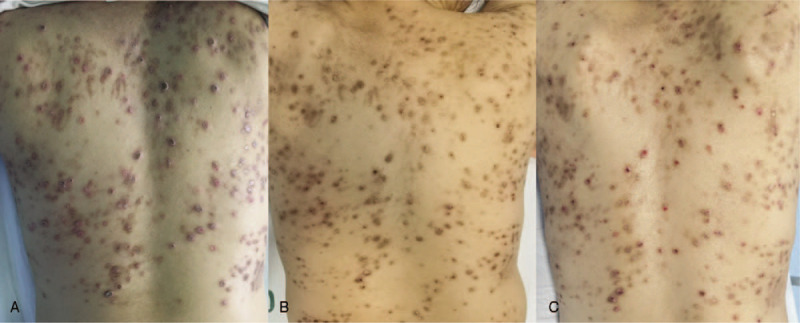
Skin lesions of the patient presented as multiple papules on back with keratotic plugs at the center on the 1st admission (A), 2nd admission (B), and 1st follow-up (C).

**Figure 2:**
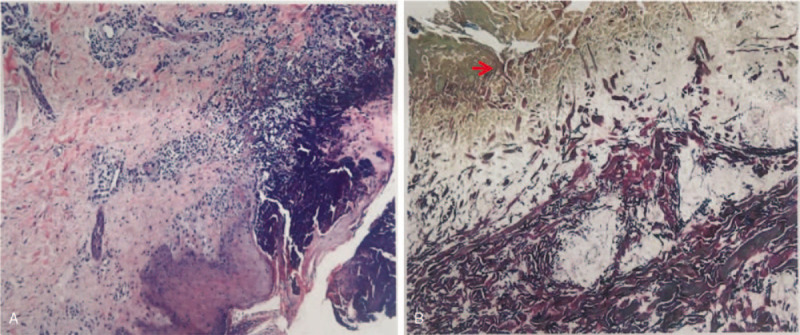
(A) Accumulation of froth histiocytes and clustered neutrophils at the base of the invagination. (B) Transepidermal penetration of collagen bundles and degeneration of elastic fibers in the superficial dermis. Positive EVG staining as indicated by the arrow.

Cutaneous examination showed diffusely distributed multiple keratotic papules on the lower limbs, buttocks, back, chest, and upper arms. The diameter of papules ranged from 2 to 8 mm, with keratotic plugs in the center. Hyperpigmentation could be observed around the papules (Fig. 1). Biopsy of the lesion from left thigh showed necrosis of squamous epithelium with the accumulation of froth histiocytes and neutrophils at the bottom of lesions. Elastic van gieson (EVG) staining was positive and collagenous fiber perforation can be observed (Fig. 2). The age of onset, the characteristic of skin lesions and histopathology met the diagnostic criteria for ARPC by Faver et al.^[3]^

This patient was treated with topical corticosteroids (halometasone cream and momeiasone furoate cream, three times a day) and oral antihistamine tablets (loratadine and ebastine, once a day) for the skin lesion and pruritus. In addition, multi-dose injection (Insulin Glargine and Aspart) together with Acarbose tablets were applied for glucose control and levothyrocine for the treatment of hypothyroidism. We used alprostadil and coated aldehyde oxystarch to improve renal function and albuminuria. During hospitalization, although the skin lesion did not respond to our local and systemic treatment, the pruritus was partially improved.

A month later, the patient was admitted to our hospital again for further treatment because the pruritus symptom was not completely relieved. In addition, although the skin lesions on the back were improved, new rashes spread to her abdomen. Laboratory examinations showed that HbA1c and renal function returned to normal but urinary microalbumin reached 3050.8 mg/L. Besides topical corticosteroids and oral antihistamine tablets, the patient was supplemented with Qingpeng ointment (including whin, benzoin, phyllanthus emblica, artificial musk, and other traditional Chinese herbs), topical retinoic acid and Zinc oxide ointment. Moreover, alprostadil and calcium dobesilate were given to correct her urinary microalbumin. On the day of hospital discharge, the keratotic plug narrowed down and pruritus relieved. Howbeit, the therapeutic effect on microalbumin was not very well.

## 4 Discussion

RPC was first described in 1967 by Mehregan.^[1]^ Though the prevalence and incidence of RPC are unknown, scattered cases have been reported. There were 10 cases of ARPC out of 5202 hospitalized patients in Bremerhaven Hospital, Germany, between 2007 and 2011.^[4]^ Investigations by Anthony Karpouzis et al indicated that the peak onset age ranged from 50 to 59 years.^[5]^

Isolated papules with keratotic plugs in the center are the characteristic skin lesions of ARPC.^[6]^ The papules could gradually develop from pinpoint size to 5 to 6 mm in diameter, with navel concave in the center. The skin lesions would relieve spontaneously, with temporary hypochromic areas and atrophic scars left. Due to various skin lesions presenting in different stages, differential diagnosis is difficult. Clinical diagnosis should be supported by histopathological features. The typical histological characteristics are the degenerated collagen bundles in the early period and thereafter the cup-shaped epidermal depression filling with keratinizing substances.^[6]^ Vertical collagen fibers are stained red by EVG staining.^[1]^

Faver et al proposed the following diagnostic criteria for ARPC:

1. transepidermal elimination of dermal connective tissue material;
2. keratotic plug at the center of umbilicated papules or nodules; and
3. the age of onset after 18 years old.^[3]^

Recently, dermoscopy is recommended as a useful and quick aid for RPC diagnosis since its diagnostic accuracy is comparable to skin biopsy and histopathology.^[7]^ In our case, keratotic plugs and perforation phenomenon could be observed and EVG staining revealed collagenous fiber perforation, which met the diagnostic criteria.

The pathogenesis of RPC has not been investigated thoroughly. The first hypothesis was presented by Mehregan, suggesting that superficial trauma may attribute to the development of RPC.^[1]^ Certain changes happen in response to injury, resulting in the intense affinity for hematoxylin in the connective tissue of the papillary layer of the corium or the wall of the superficial capillaries. Subsequently, the parakeratosis and atrophy of epidermis with transepidermal elimination of collagen occur.^[1]^ Kurschat et al proposed that scratching due to pruritus may be one of the main causes that trigger the development of RPC.^[8]^ Accordingly, we consider that superficial skin trauma caused by scratching appears to play a critical part in the pathogenesis of RPC.

It has been known that ARPC is associated with various systemic diseases. Amongst, diabetes,^[3,6,9–29]^ CRF,^[3,10–16,18,19,23,24,26–28,30–32]^ and hypertension^[3,10–13,15,26]^ are the most common coexisting disorders (Table 2). Other systemic diseases are also reported in patients with ARPC, including a range of renal,^[3,21,23]^ cardiovascular,^[3,8,10,13,15,29]^ respiratory,^[3,12,15,21,33]^ digestive,^[3,10,12–15]^ hematological,^[3,15]^ autoimmune,^[10,33–40]^ vascular,^[3,13–15]^ and skin disorders.^[3,8,12,21,22,41]^ (Table 2) Malignant neoplasms contribute to the development of ARPC as well.^[3,9,11,13,21,25,42–47]^ Rarely, ARPC occurs along with hypo- or hyperthyroidism.^[3,12]^ Pregnancy has also been reported in conjunction with the onset of ARPC.^[12,35]^ The patient in this case was complicated with T2DM, CRF, and hypothyroidism. To the best of our knowledge, ARPC with these three different systemic diseases has not been reported previously.

**Table 2:**
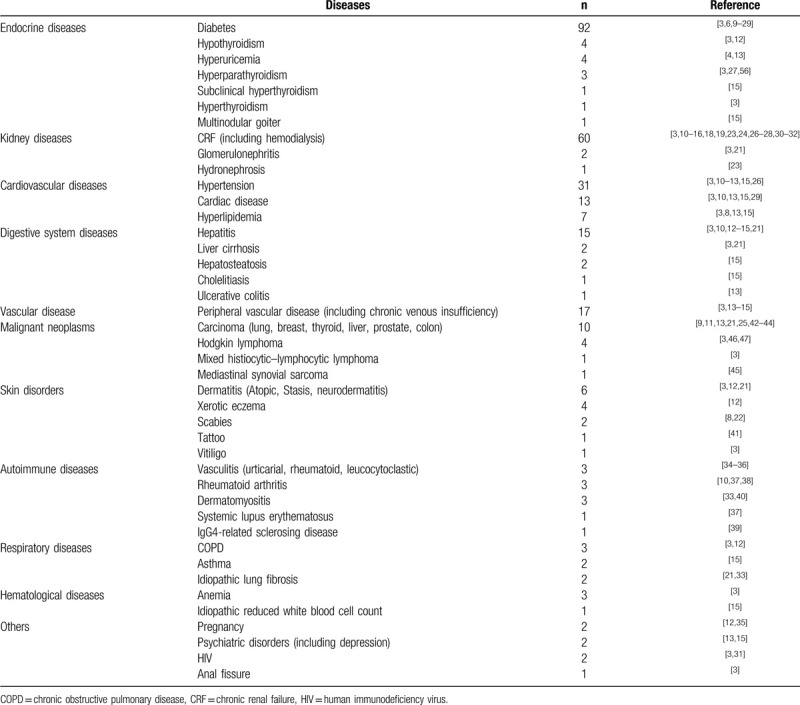
Acquired reactive perforating collagenosis associated systemic diseases.

There are several studies that identified the potential association of diabetic microangiopathy with ARPC.^[15,16,48]^ Hypoxic state may cause the separation of collagen and disruption of inter keratinocyte. Accordingly, it speculated that diabetic microvasculopathy and the resultant hypoxic state in the dermis may be a predisposing factor of ARPC.^[12]^

In addition, some crystal-like micro-deposits in the upper dermis have been observed in one ultrastructural study.^[49]^ Thus, micro-deposition of certain substances, such as calcium salts, uric acid or hydroxyapatite, in the dermis due to metabolic disorders (e.g., hyperparathyroidism, hyperuricemia, etc) may promote local inflammatory reactions and connective tissue degradation.^[13,20]^

Furthermore, the receptor for advanced glycation end products (RAGE) is a multiligand transmembrane receptor that plays an important role in inflammatory responses. Akoglu et al found that RAGE expression in microvascular endothelium, inflammatory cells and fibroblasts of patients with ARPC was more intense than normal tissues of healthy participants, indicating that RAGE may be involved in the pathogenesis of ARPC.^[50]^

There are no standardized therapeutic strategies or guidelines for ARPC by now. The management of associated internal or oncological diseases is necessary for effective dermatological treatment of ARPC.^[9,25,43]^ It was reported that, among 10 cases with cancers, PD resolved in 7 patients after successful therapy for malignancy.^[43]^ In addition, Kim et al reported a female patient with uncontrolled diabetes mellitus and metastatic breast carcinoma. Without any dermatologic interventions, the lesions of this patient were significantly improved after 10-month therapies on cancer and glycemic control.^[9]^

The treatment regimens for pruritus and skin lesions are varied. We systemically summarize the therapeutic measures and their efficacy of ARPC in Table 3 . Either single or combined therapies have been reported. Of note, only 23.8% of reported cases were treated with single therapeutic measure. Monotherapy includes topical steroid/retinoic acid, oral antibiotics (e.g., minocycline, roxithromycin, etc), diaphenyl sulfone, tranilast, and allopurinol.^[3,12,17,21,51,52]^ The effect of allopurinol is contradictory. In a few patients without hyperuricemia, allopurinol therapy resulted in the improvement of ARPC.^[23,53]^ It is known that allopurinol is an inhibitor of xanthine oxidasen which could decrease collagen damages by eliminating oxygen free radicals and inhibiting collagen cross-linking mediated by advanced glycation end products (AGE).^[27,54]^ This may explain the underlying mechanism of allopurinol in the treatment of ARPC.^[26]^ Triamcinolone intralesional injection (TA ILI) is also applied in some cases and the skin lesions in all these patients were improved.^[12]^ Phototherapy is another option for pruritus. Patients with ARPC usually receive narrow-band ultraviolet B (NB-UVB) therapy, which may have direct anti-inflammatory and anti-proliferative effects.^[3,12,15,30,32,50]^ Transcutaneous electrical nerve stimulation (TENS) can be safe and effective, especially for patients with severe itch.^[55]^

**Table 3:**
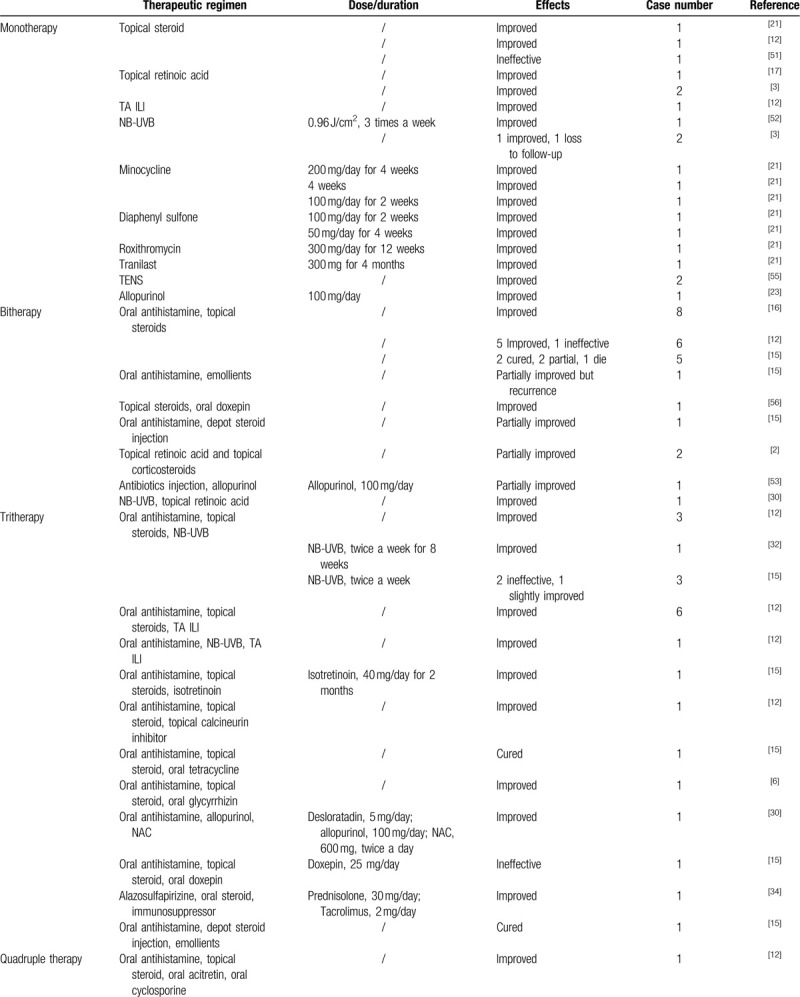
Dermatological treatment for acquired reactive perforating collagenosis.

**Table 3 (Continued):**
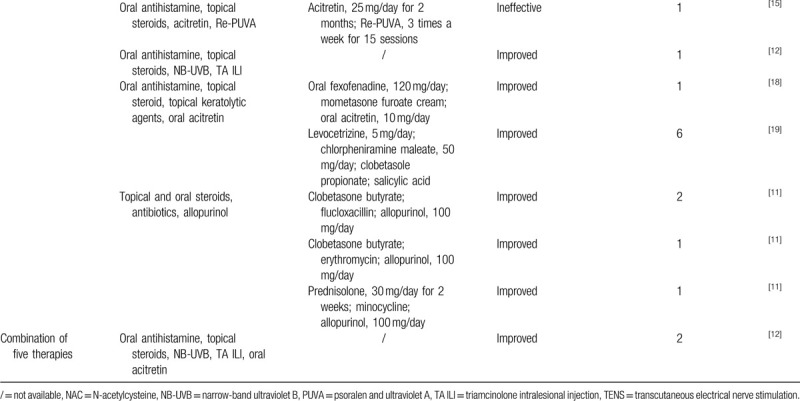
Dermatological treatment for acquired reactive perforating collagenosis.

There were 31.0% cases treated with synergetic bitherapy. Widely employed remedies are oral antihistamines and topical steroids^[12,15,16]^, which can also be respectively combined with emollients^[15]^ or oral doxepin.^[56]^ As shown in Table 3 , almost all tri- and quadruple therapies include oral antihistamines and topical steroids. There is only one study that reports the combination of five therapies for ARPC treatment,^[12]^ which includes oral antihistamine, topical steroids, NB-UVB, TA ILI, and oral acitretin.

Although there are various therapeutic measures, the curative effect is not satisfactory. As summarized in Table 3 , symptoms of most reported cases can only be improved rather than cured. In addition, the duration for ARPC treatment is long, ranging from 2 weeks to 4 months. In this case, although bitherapy of oral antihistamine and topical steroids could partially relieve pruritus at first, the symptoms were not completely relieved after 1 month treatment. Supplementary therapy including topical retinoic acid, Qingpeng ointment, and Zinc oxide ointment were employed on her second admission and the pruritus and skin lesions were significantly improved. Accordingly, we recommend that ARPC can be managed by co-administration of two or more agents. Howbeit, the synergetic mechanism of these agents is unclear.

## 5 Conclusion

We reported a rare case of ARPC associated with three systemic diseases including T2DM, CRF, and hypothyroidism. Recognition and control of concomitant diseases may help to treat ARPC. Dermatological treatment of pruritus and skin lesions is featured by the application of a wide range of regimens. Nevertheless, the response to these therapeutic measures is uncertain. Further exploration in this field is necessitated.

## Author contributions

**Conceptualization:** Shiying Shao.

**Data curation:** Xinyue Zhang, Yan Yang.

**Investigation:** Yan Yang.

**Validation:** Yan Yang.

**Writing – original draft:** Xinyue Zhang.

**Writing – review & editing:** Shiying Shao.
